# Supplementary material for: Alterations of the Platelet Proteome in Lung Cancer: Accelerated F13A1 and ER Processing as New Actors in Hypercoagulability
Source: Cancers (Basel). 2021 May 8;13(9):2260. doi: 10.3390/cancers13092260 (PMC8125802; doi:10.3390/cancers13092260)
Supplement: Supplementary file 1 [file cancers-13-02260-s001.zip › cancers-1216936-supplementary-for pub/cancers-1216936- supplementary materials.pdf]

cancers

Supplementary Materials: Alterations of the Platelet Proteome in Lung Cancer: Accelerated F13A1 and ER Processing As New Actors in Hypercoagulability

Huriye Ercan, Lisa-Marie Mauracher, Ella Grilz, Lena Hell, Roland Hellinger, Johannes A. Schmid, Florian Moik, Cihan Ay, Ingrid Pabinger and Maria Zellner

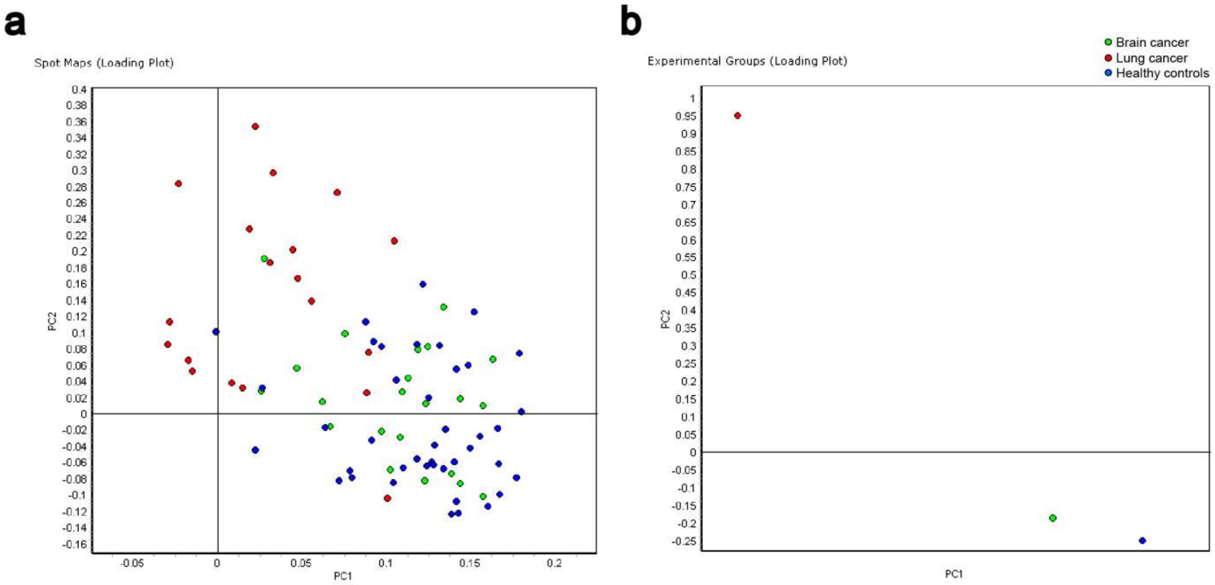

Figure S1. Principle component analysis of variable platelet proteins between controls, brain and lung cancer patients. PCA plot analysis is based on 2D-DIGE analysis and one-way ANOVA filtered platelet protein spot changes between the three study groups. (**a**) Dots represent the particular platelet samples aggregated from 36 one-way ANOVA-based significant different platelet protein spots from 22 patients with brain cancer, 19 patients with lung cancer and 41 age and sex matched healthy controls. (**b**) Reduction of all platelet samples from each study group to one value to illustrate simplified the influence of cancer types compared to healthy controls from the acidic platelet proteome (pH range 4-7). Abbreviations: PCA - Principle component analysis, PC1 – Principal Component 1; PC2 – Principal Component 2.

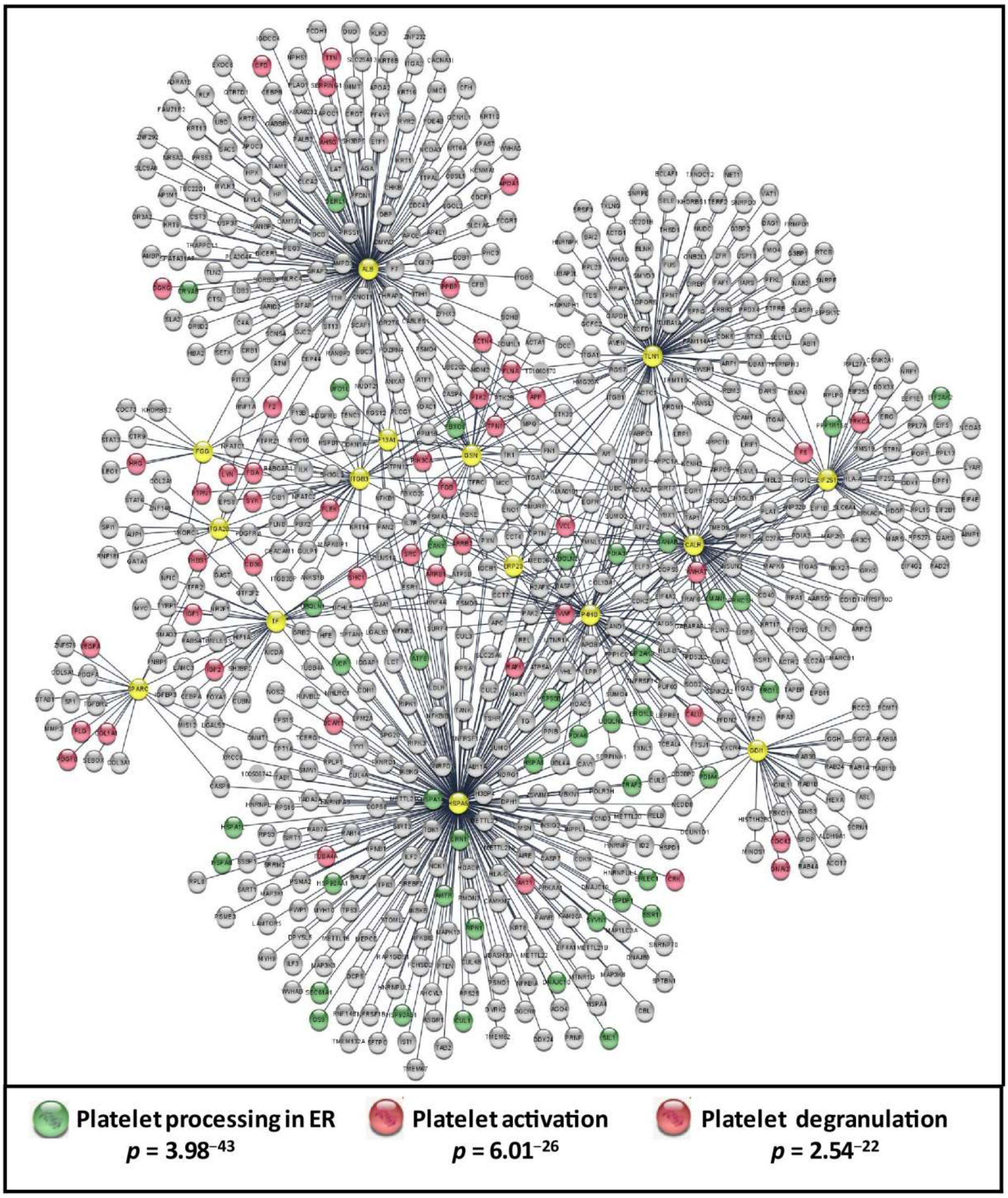

Figure S2. Alternative functional association analysis of lung cancer-related platelet proteins by NetworkAnalyst. Minimally-connected first-order integrated protein-protein interaction network of the lung-cancer related platelet proteins. The comparison of the Interactome with the Reactome database and the comparison with KEGG are shown. The seed-nodes (2D-DIGE characterized lung cancer related platelet proteins) are labelled in yellow Network analysis of proteomics data showed similar enriched pathways as revealed by STRING database analysis. Furthermore, NetworkAnalyst demonstrates many additionally known as well as novel nodes of these pathways.

a F13A1 spot 11

2 10 20 30 40 50 60 70 80 90 100
SETSRTAFG GRRAVPPNNS NAAEDDLPTV ELQGVVPRGV NLQEFLNVTS VHLFKERWDT NKVDHHTDKY ENNKLIVRRG QSFYVQIDFS RPYDPRRDLF
110 120 130 140 150 160 170 180 190 200
RVEYVIGRYP QENKGTYIPV PIVSELQSGK WGAKIVMRED RSVRLSIQSS PKCIVGKFRM YVAVWTPYGV LRTSRNPETD TYILFNPWCE DDAVYLDNEK
210 220 230 240 250 260 270 280 290 300
EREEYVLNDI GVIFYGEVND IKTRSWSYGQ FEDGILDTCL YVMDRAQMDL SGRGNPIKVS RVGSAMVNAK DDEGVLVGSW DNIYAYGVPP SAWTGSVDIL
310 320 330 340 350 360 370 380 390 400
LEYRSSENPV RYGQCWVFAG VFNTFLRCLG IPARIVTNYF SAHDNDANLQ MDIFLEEDGN VNSKLTKDSV WNYHCWNEAW MTRPDLPVGF GGWQAVDSTP
410 420 430 440 450 460 470 480 490 500
QENSDGMYRC GPASVQAIKH GHVCFQFDAP FVFAEVNSDL IYITAKKDGT HVVENVDATH IGKLIVTKQI GGDGMMDITD TYKFQEGQEE ERLALETALM
510 520 530 540 550 560 570 580 590 600
YGAKKPLNTE GVMKSRSNVD MDFEVENAVL GKDFKLSITF RNNSHNRYTI TAYLSANITF YTGVPKAEFK KETFDVTLEP LSFKKEAVLI QAGEYMGQLL
610 620 630 640 650 660 670 680 690 700
EQASLHFFVT ARINETRDVL AKQKSTVLTI PEIIIKVRGT QVVGSDMTVT VQFTNPLKET LRNVWVHLDG PGVTRPMKKM FREIRPNSTV QWEEVCRPWV
710 720 730
SGHRKLIASM SSDSLRHVYG ELDVQIQRRP SM

b

Activation peptide	S2 – R38
Active sites (catalytic triad)	C315, H374, D397
Ca^{2+}-binding sites	N437, D439, E486, E491
Potential Plasmin inactivation cleavage sites	K468 , Q469, R492, K570
Thrombin inactivation cleavage site	K514
Chymase inactivation cleavage site	F574

c F13A1 spot 12

2 10 20 30 40 50 60 70 80 90 100
SETSRTAFG GRRAVPPNNS NAAEDDLPTV ELQGVVPRGV NLQEFLNVTS VHLFKERWDT NKVDHHTDKY ENNKLIVRRG QSFYVQIDFS RPYDPRRDLF
110 120 130 140 150 160 170 180 190 200
RVEYVIGRYP QENKGTYIPV PIVSELQSGK WGAKIVMRED RSVRLSIQSS PKCIVGKFRM YVAVWTPYGV LRTSRNPETD TYILFNPWCE DDAVYLDNEK
210 220 230 240 250 260 270 280 290 300
EREEYVLNDI GVIFYGEVND IKTRSWSYGQ FEDGILDTCL YVMDRAQMDL SGRGNPIKVS RVGSAMVNAK DDEGVLVGSW DNIYAYGVPP SAWTGSVDIL
310 320 330 340 350 360 370 380 390 400
LEYRSSENPV RYGQCWVFAG VFNTFLRCLG IPARIVTNYF SAHDNDANLQ MDIFLEEDGN VNSKLTKDSV WNYHCWNEAW MTRPDLPVGF GGWQAVDSTP
410 420 430 440 450 460 470 480 490 500
QENSDGMYRC GPASVQAIKH GHVCFQFDAP FVFAEVNSDL IYITAKKDGT HVVENVDATH IGKLIVTKQI GGDGMMDITD TYKFQEGQEE ERLALETALM
510 520 530 540 550 560 570 580 590 600
YGAKKPLNTE GVMKSRSNVD MDFEVENAVL GKDFKLSITF RNNSHNRYTI TAYLSANITF YTGVPKAEFK KETFDVTLEP LSFKKEAVLI QAGEYMGQLL
610 620 630 640 650 660 670 680 690 700
EQASLHFFVT ARINETRDVL AKQKSTVLTI PEIIIKVRGT QVVGSDMTVT VQFTNPLKET LRNVWVHLDG PGVTRPMKKM FREIRPNSTV QWEEVCRPWV
710 720 730
SGHRKLIASM SSDSLRHVYG ELDVQIQRRP SM

d F13A1 spot 12a

2 10 20 30 40 50 60 70 80 90 100
SETSRTAFG GRRAVPPNNS NAAEDDLPTV ELQGVVPRGV NLQEFLNVTS VHLFKERWDT NKVDHHTDKY ENNKLIVRRG QSFYVQIDFS RPYDPRRDLF
110 120 130 140 150 160 170 180 190 200
RVEYVIGRYP QENKGTYIPV PIVSELQSGK WGAKIVMRED RSVRLSIQSS PKCIVGKFRM YVAVWTPYGV LRTSRNPETD TYILFNPWCE DDAVYLDNEK
210 220 230 240 250 260 270 280 290 300
EREEYVLNDI GVIFYGEVND IKTRSWSYGQ FEDGILDTCL YVMDRAQMDL SGRGNPIKVS RVGSAMVNAK DDEGVLVGSW DNIYAYGVPP SAWTGSVDIL
310 320 330 340 350 360 370 380 390 400
LEYRSSENPV RYGQCWVFAG VFNTFLRCLG IPARIVTNYF SAHDNDANLQ MDIFLEEDGN VNSKLTKDSV WNYHCWNEAW MTRPDLPVGF GGWQAVDSTP
410 420 430 440 450 460 470 480 490 500
QENSDGMYRC GPASVQAIKH GHVCFQFDAP FVFAEVNSDL IYITAKKDGT HVVENVDATH IGKLIVTKQI GGDGMMDITD TYKFQEGQEE ERLALETALM
510 520 530 540 550 560 570 580 590 600
YGAKKPLNTE GVMKSRSNVD MDFEVENAVL GKDFKLSITF RNNSHNRYTI TAYLSANITF YTGVPKAEFK KETFDVTLEP LSFKKEAVLI QAGEYMGQLL
610 620 630 640 650 660 670 680 690 700
EQASLHFFVT ARINETRDVL AKQKSTVLTI PEIIIKVRGT QVVGSDMTVT VQFTNPLKET LRNVWVHLDG PGVTRPMKKM FREIRPNSTV QWEEVCRPWV
710 720 730
SGHRKLIASM SSDSLRHVYG ELDVQIQRRP SM

e F13A1 spot 12b

2 10 20 30 40 50 60 70 80 90 100
SETSRTAFG GRRAVPPNNS NAAEDDLPTV ELQGVVPRGV NLQEFLNVTS VHLFKERWDT NKVDHHTDKY ENNKLIVRRG QSFYVQIDFS RPYDPRRDLF
110 120 130 140 150 160 170 180 190 200
RVEYVIGRYP QENKGTYIPV PIVSELQSGK WGAKIVMRED RSVRLSIQSS PKCIVGKFRM YVAVWTPYGV LRTSRNPETD TYILFNPWCE DDAVYLDNEK
210 220 230 240 250 260 270 280 290 300
EREEYVLNDI GVIFYGEVND IKTRSWSYGQ FEDGILDTCL YVMDRAQMDL SGRGNPIKVS RVGSAMVNAK DDEGVLVGSW DNIYAYGVPP SAWTGSVDIL
310 320 330 340 350 360 370 380 390 400
LEYRSSENPV RYGQCWVFAG VFNTFLRCLG IPARIVTNYF SAHDNDANLQ MDIFLEEDGN VNSKLTKDSV WNYHCWNEAW MTRPDLPVGF GGWQAVDSTP
410 420 430 440 450 460 470 480 490 500
QENSDGMYRC GPASVQAIKH GHVCFQFDAP FVFAEVNSDL IYITAKKDGT HVVENVDATH IGKLIVTKQI GGDGMMDITD TYKFQEGQEE ERLALETALM
510 520 530 540 550 560 570 580 590 600
YGAKKPLNTE GVMKSRSNVD MDFEVENAVL GKDFKLSITF RNNSHNRYTI TAYLSANITF YTGVPKAEFK KETFDVTLEP LSFKKEAVLI QAGEYMGQLL
610 620 630 640 650 660 670 680 690 700
EQASLHFFVT ARINETRDVL AKQKSTVLTI PEIIIKVRGT QVVGSDMTVT VQFTNPLKET LRNVWVHLDG PGVTRPMKKM FREIRPNSTV QWEEVCRPWV
710 720 730
SGHRKLIASM SSDSLRHVYG ELDVQIQRRP SM

f F13A1 spot 12c

2 10 20 30 40 50 60 70 80 90 100
SETSRTAFG GRRAVPPNNS NAAEDDLPTV ELQGVVPRGV NLQEFLNVTS VHLFKERWDT NKVDHHTDKY ENNKLIVRRG QSFYVQIDFS RPYDPRRDLF
110 120 130 140 150 160 170 180 190 200
RVEYVIGRYP QENKGTYIPV PIVSELQSGK WGAKIVMRED RSVRLSIQSS PKCIVGKFRM YVAVWTPYGV LRTSRNPETD TYILFNPWCE DDAVYLDNEK
210 220 230 240 250 260 270 280 290 300
EREEYVLNDI GVIFYGEVND IKTRSWSYGQ FEDGILDTCL YVMDRAQMDL SGRGNPIKVS RVGSAMVNAK DDEGVLVGSW DNIYAYGVPP SAWTGSVDIL
310 320 330 340 350 360 370 380 390 400
LEYRSSENPV RYGQCWVFAG VFNTFLRCLG IPARIVTNYF SAHDNDANLQ MDIFLEEDGN VNSKLTKDSV WNYHCWNEAW MTRPDLPVGF GGWQAVDSTP
410 420 430 440 450 460 470 480 490 500
QENSDGMYRC GPASVQAIKH GHVCFQFDAP FVFAEVNSDL IYITAKKDGT HVVENVDATH IGKLIVTKQI GGDGMMDITD TYKFQEGQEE ERLALETALM
510 520 530 540 550 560 570 580 590 600
YGAKKPLNTE GVMKSRSNVD MDFEVENAVL GKDFKLSITF RNNSHNRYTI TAYLSANITF YTGVPKAEFK KETFDVTLEP LSFKKEAVLI QAGEYMGQLL
610 620 630 640 650 660 670 680 690 700
EQASLHFFVT ARINETRDVL AKQKSTVLTI PEIIIKVRGT QVVGSDMTVT VQFTNPLKET LRNVWVHLDG PGVTRPMKKM FREIRPNSTV QWEEVCRPWV
710 720 730
SGHRKLIASM SSDSLRHVYG ELDVQIQRRP SM

g F13A1 spot 12d

2 10 20 30 40 50 60 70 80 90 100
SETSRTAFG GRRAVPPNNS NAAEDDLPTV ELQGVVPRGV NLQEFLNVTS VHLFKERWDT NKVDHHTDKY ENNKLIVRRG QSFYVQIDFS RPYDPRRDLF
110 120 130 140 150 160 170 180 190 200
RVEYVIGRYP QENKGTYIPV PIVSELQSGK WGAKIVMRED RSVRLSIQSS PKCIVGKFRM YVAVWTPYGV LRTSRNPETD TYILFNPWCE DDAVYLDNEK
210 220 230 240 250 260 270 280 290 300
EREEYVLNDI GVIFYGEVND IKTRSWSYGQ FEDGILDTCL YVMDRAQMDL SGRGNPIKVS RVGSAMVNAK DDEGVLVGSW DNIYAYGVPP SAWTGSVDIL
310 320 330 340 350 360 370 380 390 400
LEYRSSENPV RYGQCWVFAG VFNTFLRCLG IPARIVTNYF SAHDNDANLQ MDIFLEEDGN VNSKLTKDSV WNYHCWNEAW MTRPDLPVGF GGWQAVDSTP
410 420 430 440 450 460 470 480 490 500
QENSDGMYRC GPASVQAIKH GHVCFQFDAP FVFAEVNSDL IYITAKKDGT HVVENVDATH IGKLIVTKQI GGDGMMDITD TYKFQEGQEE ERLALETALM
510 520 530 540 550 560 570 580 590 600
YGAKKPLNTE GVMKSRSNVD MDFEVENAVL GKDFKLSITF RNNSHNRYTI TAYLSANITF YTGVPKAEFK KETFDVTLEP LSFKKEAVLI QAGEYMGQLL
610 620 630 640 650 660 670 680 690 700
EQASLHFFVT ARINETRDVL AKQKSTVLTI PEIIIKVRGT QVVGSDMTVT VQFTNPLKET LRNVWVHLDG PGVTRPMKKM FREIRPNSTV QWEEVCRPWV
710 720 730
SGHRKLIASM SSDSLRHVYG ELDVQIQRRP SM

Figure S3. Amino acid sequence coverage from F13A1 protein spots identifications by MS analysis. The amino acid sequence of the mature F13A1 protein is accessed from Uniprot database (https://www.uniprot.org/uniprot/P00488). (**a**) The peptides detected by MS analysis from tryptic digested 55 kDa F13A1 spot 11 are underlined. The activation peptide is highlighted in green, active sites in red, Ca^{2+}-binding sites in blue, thrombin inactivation site in orange, plasmin cut sites in purple and chymase inactivation site in pink. (**b**) Compilation of important functional positions from F13A1. Further amino acid sequence coverage from F13A1 identification by MS analysis are specified from (**c**) spot 12, (**d**) F13A1 spot 12a, (**e**) F13A1 spot 12b, (**f**) F13A1 spot 12c, and (**g**) F13A1 spot 12d.

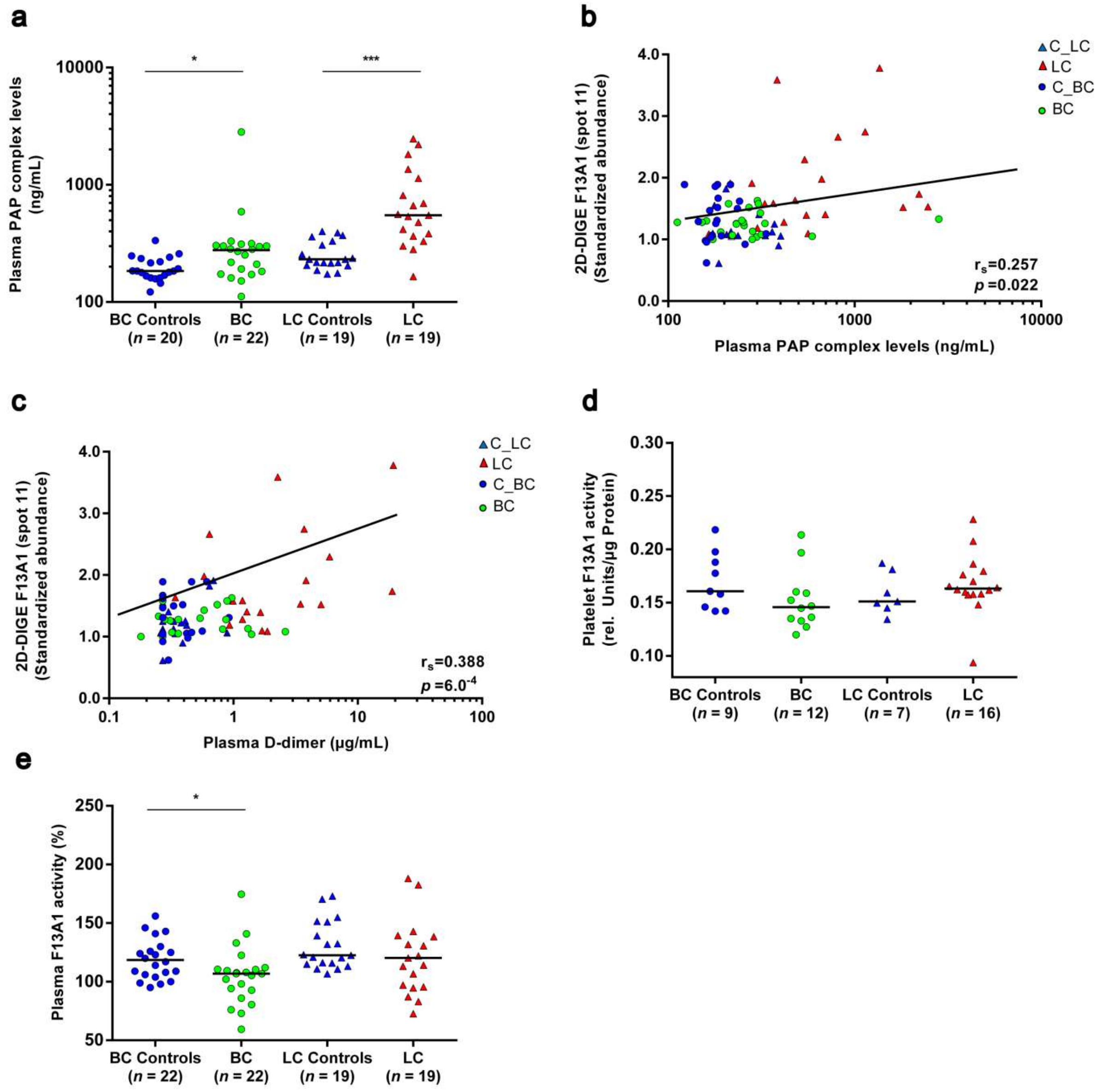

Figure S4. Correlation of the 55 kDa fragment abundance from F13A1 in platelets with plasma fibrinolysis marker and F13A1 enzymatic activity in platelets and plasma of the study cohorts. (**a**) PAP complex levels in plasma. (**b**) Scatter dot plot and correlation analysis of 55 kDa F13A1 standardized platelet protein spot abundances quantified by 2D-DIGE with corresponding plasma PAP complex levels and (**c**) plasma D-dimer levels. An association was assessed by a Spearman´s Rank correlation coefficient (r_s). (**d**) F13A1 activity levels in platelets and (**e**) plasma in patients with lung and brain cancer and matched healthy controls. Protein levels were depicted as single values and median. * p <0.05, ** p <0.01, *** p <0.001. Abbreviations: 2D-DIGE – two-dimensional differential in-gel electrophoresis; LC – patient with lung cancer; BC – patient with brain cancer; C – healthy control; PAP – plasmin-α-2-antiplasmin.

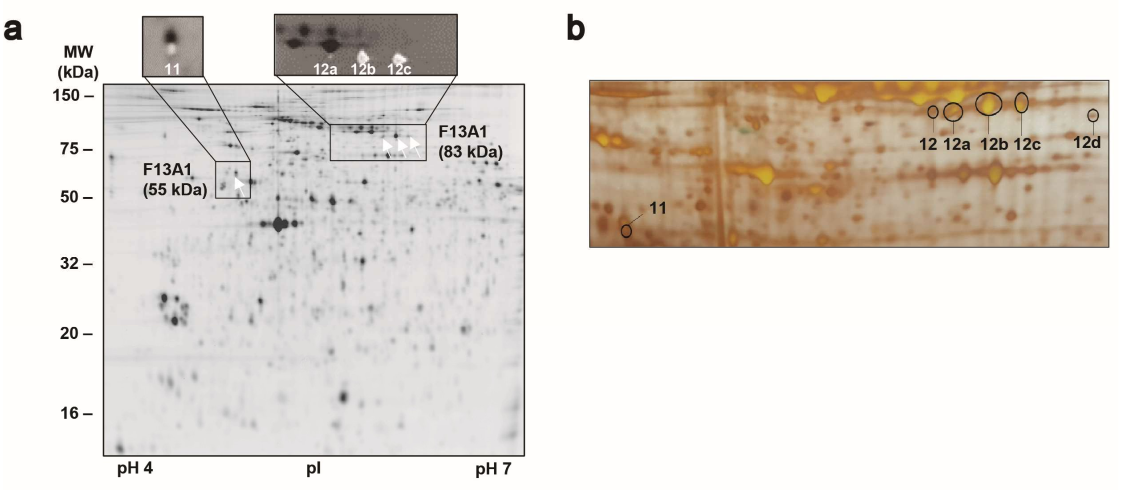

Figure S5. Identified proteoforms of F13A1 in the platelet proteome. (**a**) 2D-DIGE image from the platelet proteome of a patient with lung cancer. (**b**) Two-dimensional gel from platelets of a patient with lung cancer stained by silver. The image b of the 2D gel was captured with a digital camera. As show in a four spots were recognized as F13A1 by 2-D WB and specific antibody, and in b additionally two F13A1 proteoforms (spot 12 and 12d) were identified by MS analysis. Detailed statistical descriptions of the highlighted proteins are listed in Table 3. Abbreviations: 2D-DIGE – two-dimensional differential in-gel electrophoresis; WB – western blot; pI – isoelectric point; MW – molecular weight; kDa – kilodalton.

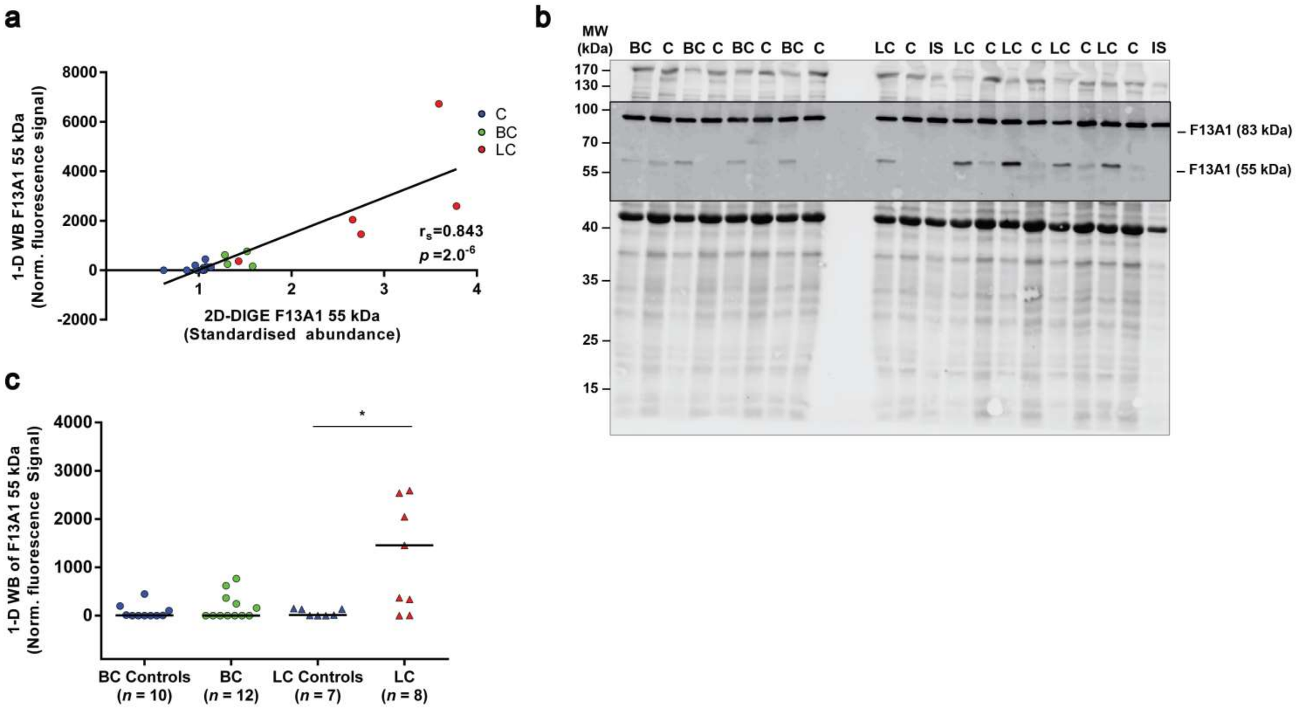

Figure S6. Validation of 2D-DIGE quantified platelet F13A1 proteoform levels by 1-D WB analysis. (**a**) Scatter dot plot and correlation analysis (Spearman´s Rank correlation coefficient) of 55 kDa F13A1 levels measured by 2D-DIGE and 1-D fluorescence WB analysis. (**b**) Representative 1-D WB image of F13A1 analysis in clinical platelet samples. Twelve µg platelet protein extract was separated per lane by a 11.5% SDS-PAGE (10 cm gel height). Ruthenium-based whole-protein stain of blotted proteins was performed to assess equal protein application. The image section shows the whole-protein stain and the overlay of the antibody signals detected by fluorescence signal (Cy5). (**c**) 1-D protein levels of 55 kDa F13A1 fragment with their medians. Abbreviations: WB: western blot; 2D-DIGE – two-dimensional differential in-gel electrophoresis; MW – molecular weight; kDa – kilodalton; C – healthy control; BC – patient with brain cancer; LC – patient with lung cancer; IS – internal standard.

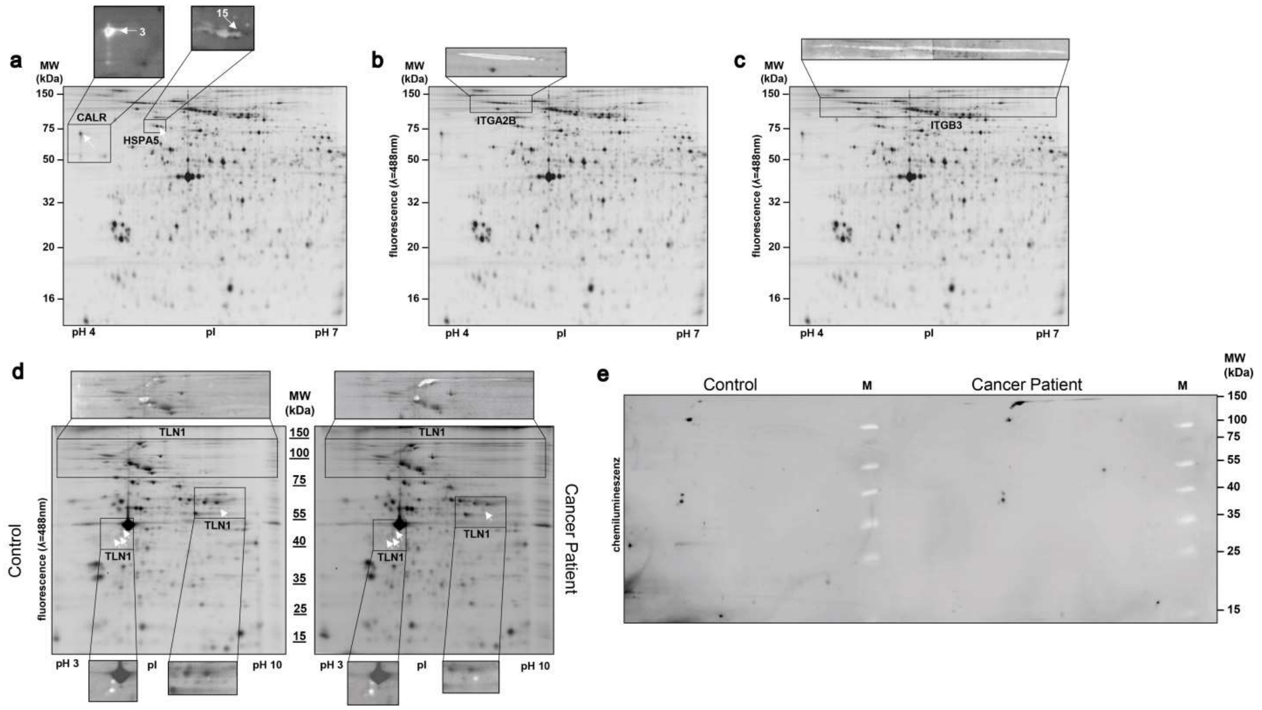

Figure S7. Two-dimensional WB validations of CALR, HSPA5, ITGA2B, ITGB3 and TLN1 MS identifications in platelet proteome. (**a**) Representative 2-D WB image of platelet CALR probed with monoclonal anti-CALR clone FMC 75 and 2-D WB image of platelet HSPA5 probed with polyclonal anti-HSPA5, (**b**) ITGA2B with polyclonal anti-CD41/ITGA2B, (**c**) ITGB3 with monoclonal anti-CD61/ ITGB3 and (**d**) TLN1 with monoclonal anti-Talin clone 8D4. Proteins of interest are highlighted with a white arrow. The cut-out region shows an overlay from Cy2-labelled 2D-DIGE gel and specific 2-D WB signals, where immune-identified proteins are displayed as white spots and Cy2-labelled spots are displayed in black. (**e**) Comparison of TLN1 signals between the control and patient sample. Abbreviations: 2D-DIGE – two-dimensional differential in-gel electrophoresis; WB – Western blot; pI – isoelectric point; M – marker; MW – molecular weight; kDa – kilodalton; λ – wavelength.

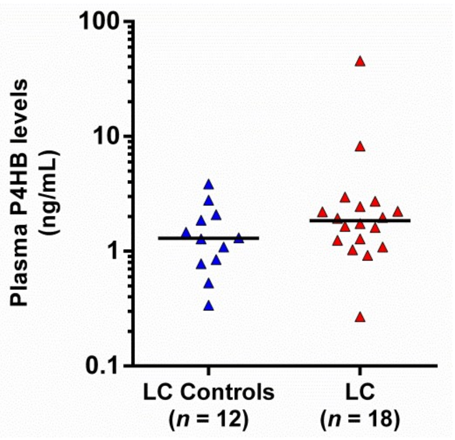

Figure S8. P4HB levels in plasma in patients with lung cancer and matched healthy controls. Each dot represents a measurement of a single patient. The line between the dots represents the median (Wilcoxon-Mann-Whitney-U-Test). Abbreviations: LC – patient with lung cancer.

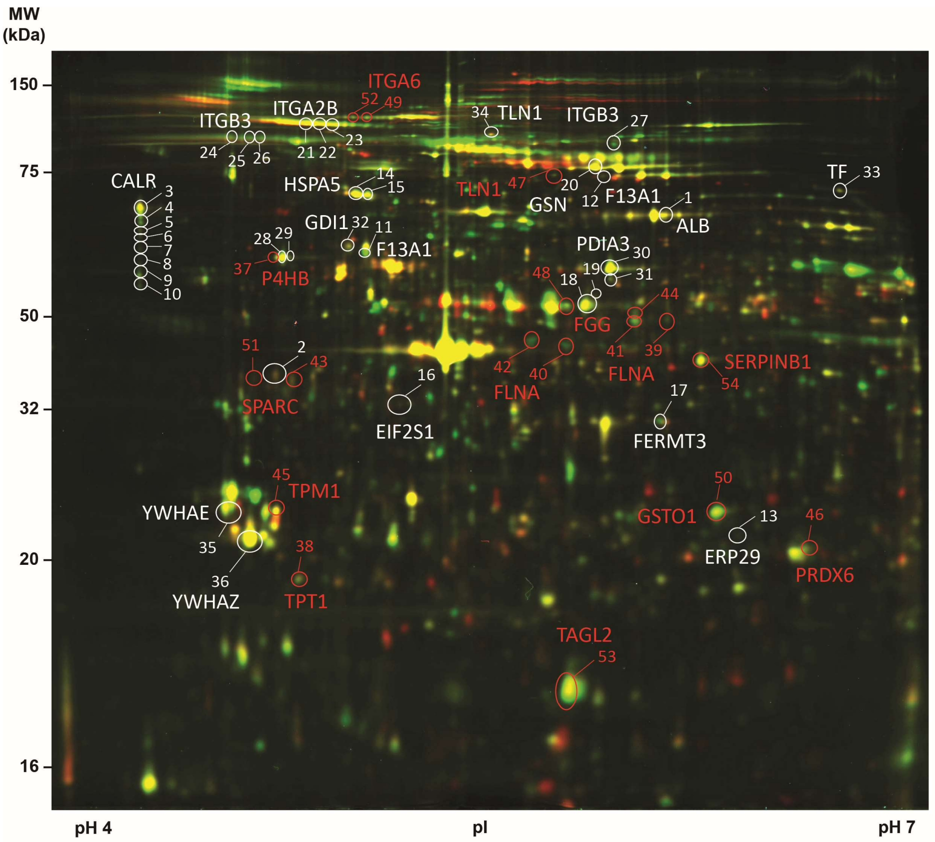

Figure S9. 2D-DIGE-based proteome analysis of platelets from patients with brain and lung cancer compared to controls. All lung cancer-related platelet protein spots of this proteomics study are indicated in this representative 2D-DIGE image. A total of 36 µg (12 µg sample Cy3-, 12 µg sample Cy5-, and 12 µg IS Cy2-labelled) platelet protein extracts were separated according to the isoelectric point (pI) in the pH 4-7 range (separation distance 24 cm) and the molecular weight (MW, separation distance 20 cm). Protein spots identified by MS are circled and labelled with their corresponding gene name and spot numbers are given in Table 1 (white) and in the Table S2 (red). Proteins equally expressed in the representative 2D-DIGE image are labelled in yellow. Only protein changes that are above a ratio of 1.5 between the samples labelled with Cy3 and Cy5 are visible in red or green. However, many of these spots that are strongly highlighted in red or green in this representative 2D-DIGE image are not significantly associated with lung cancer, but rather seem to stand out due to the biological variation of the respective individuals. Abbreviations: MW – molecular weight; kDa – kilodalton; pI – isoelectric point; 2D-DIGE – two-dimensional differential in-gel electrophoresis; IS – internal standard; MS – mass spectrometry.

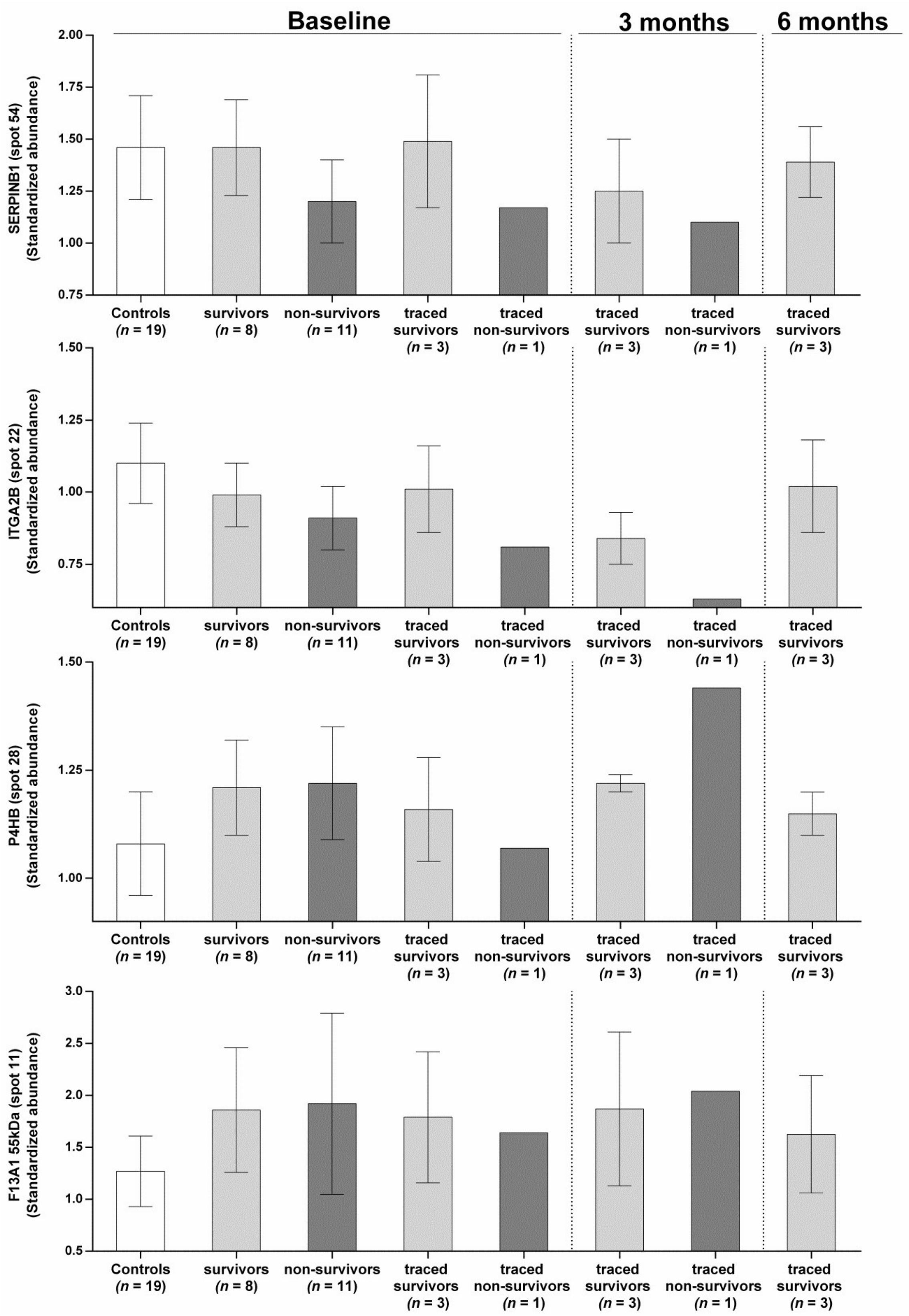

Figure S10. Follow up platelet proteomics analysis of patients with lung cancer. The platelet protein levels of SERPINB1, ITGA2B, P4HB, 55 kDa F13A1 are specified as standardized abundance from 2D-DIGE (pH 4-7) analysis. All values are depicted as mean ± standard deviation. The four traced lung cancer patients came from the total patient group with lung cancer.

Table S1. Influence of pI and MW-modifying PTMs on abundance of F13A1 proteoforms in clinical platelet samples. Correlations of F13A1 spot 12c abundance to other proteoforms are calculated explorative by Spearman correlation coefficients with corresponding *p*-values from patients with lung cancer (*n* = 19) and brain cancer (*n* = 22) and all matched controls (*n* = 41). Abbreviations: pI – isoelectric point; MW – molecular weight; PTM – post translational modification.

	**Enzymatic inactive F13A1 (12c)**	
**Protein name**	**Spearman´s rho**	***p*-value**
**F13A1 (12b)**	-0.520	1.05E-06
**F13A1 (12)**	-0.393	3.13E-04
**F13A1 (12a)**	-0.377	5.63E-04
**F13A1 (12d)**	-0.193	9.47E-02
**F13A1 (11)**	-0.084	4.67E-01
**F13A1 (12b)**	-0.520	1.05E-06

Table S2. List of identified proteins. Addition to Table 1/or Figure 6 with further lung-cancer related platelet proteoforms and/or platelet proteoforms significantly correlating with haemostatic plasma proteins. Platelet protein profiles are analysed by 2D-DIGE and platelet protein levels are given as standardised abundance. Statistical data are specified between patients with brain cancer (*n* = 22), lung cancer (*n* = 19) and healthy controls (*n* = 41). Spot numbering continued from Table 1. The protein spots are shown in Figure S9. Abbreviations: MW – molecular weight; Da – dalton; pI – isoelectric point; FC – fold change.

						**Brain cancer patients / Healthy controls**			**Lung cancer patients / Healthy controls**		
**Spot number**	**Protein name**	**Gene name**	**Isoelectric point (pI)**	**MW (Da)**	**One-way ANOVA (adjusted)**	**Average FC**	***p*-value (unadjusted)**	***p*-value (adjusted)**	**Average FC**	***p*-value (unadjusted)**	***p*-value (adjusted)**
37	Protein disulfide-isomerase	P4HB	4.76	61026	0.2310	1.06	0.4631	0.8548	1.09	0.0787	0.0910
38	Translationally-controlled tumor protein	TPT1	4.84	19595	0.1790	1.00	0.9888	0.9888	1.09	0.0470	0.0603
39	Filamin-A	FLNA	5.77	51897	0.0675	0.94	0.4926	0.8548	0.76	0.0008	0.0043
40	Filamin-A	FLNA	5.51	50066	0.0962	0.92	0.4443	0.8548	0.67	0.0012	0.0054
41	Filamin-A	FLNA	5.68	51897	0.1790	0.95	0.5979	0.8819	0.73	0.0004	0.0034
42	Filamin-A	FLNA	5.41	50066	0.0675	0.93	0.4809	0.8548	0.70	0.0019	0.0062
43	Basement-membrane protein 40 (SPARC; Osteonectin)	SPARC	4.82	42014	0.1000	0.96	0.6705	0.8864	0.67	0.0001	0.0015
44	Filamin-A	FLNA	5.68	52656	0.1090	0.99	0.8614	0.9350	0.73	0.0014	0.0059
45	Tropomyosin alpha-1 chain	TPM1	4.77	30066	0.3110	1.03	0.7961	0.9350	0.72	0.0012	0.0054
46	Peroxiredoxin-6	PRDX6	6.48	26537	0.3590	0.96	0.4491	0.8548	0.88	0.0167	0.0246
47	Talin-1	TLN1	5.47	71098	0.1310	0.92	0.5709	0.8819	0.67	0.0144	0.0236
48	Fibrinogen gamma chain	FGG	5.50	55042	0.0517	0.99	0.8766	0.9350	1.28	0.0004	0.0034
49	Integrin alpha-6	ITGA6	5.09	126605	0.2250	0.94	0.2969	0.7616	0.89	0.0225	0.0309
50	Glutathione S-transferase omega-1	GSTO1	5.89	29925	0.6150	1.05	0.7374	0.9064	0.79	0.0842	0.0955

51	Basement-membrane protein 40 (SPARC; Osteonectin)	SPARC	4.71	42014	0.0828	0.94	0.5357	0.8819	0.71	0.0008	0.0043
52	Integrin alpha-6	ITGA6	5.02	126605	0.2500	0.97	0.6485	0.8864	0.91	0.1609	0.1695
53	Transgelin-2	TAGL2	5.51	8188	0.1190	0.96	0.6222	0.8864	0.82	0.0117	0.0223
54	Leukocyte elastase inhibitor	SERPINB1	5.9	42741	0.1310	0.93	0.1387	0.6781	0.87	0.0152	0.0236
