# Supplementary material for: Alterations of the Platelet Proteome in Lung Cancer: Accelerated F13A1 and ER Processing as New Actors in Hypercoagulability
Source: Cancers (Basel). 2021 May 8;13(9):2260. doi: 10.3390/cancers13092260 (PMC8125802; doi:10.3390/cancers13092260)
Supplement: Supplementary file 1 [file cancers-13-02260-s001.zip › cancers-1216936-supplementary-for pub/Ercan et al Westernblots.pdf]

Supplementary Materials:

Alterations of the Platelet Proteome in Lung Cancer: Accelerated F13A1 and ER Processing As New Actors in Hypercoagulability

Huriye Ercan, Lisa-Marie Mauracher, Ella Grilz, Lena Hell, Roland Hellinger, Johannes A. Schmid, Florian Moik, Cihan Ay, Ingrid Pabinger, Maria Zellner

(**a**)

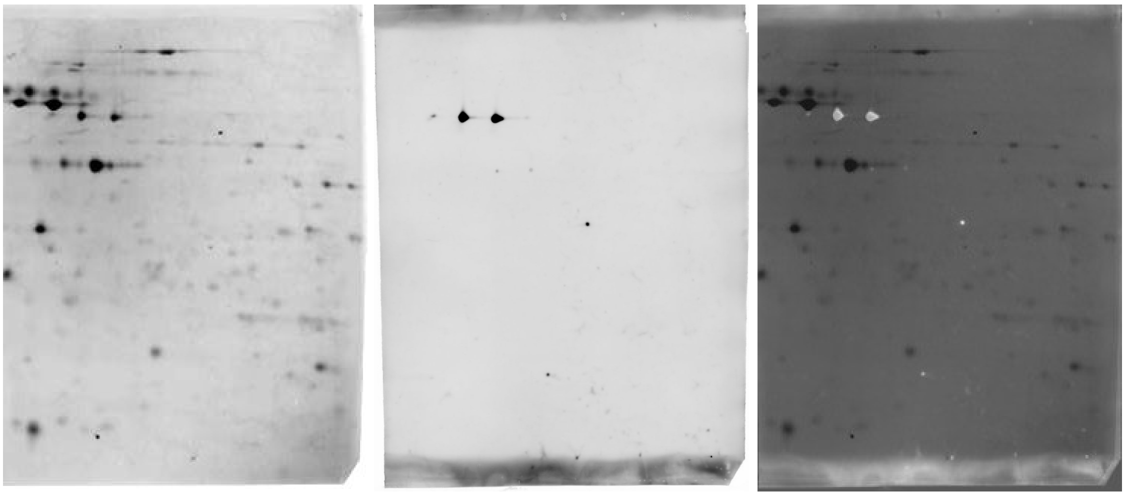

Cy2 signal **Cy5 signal** **Overlay**

(**b**)

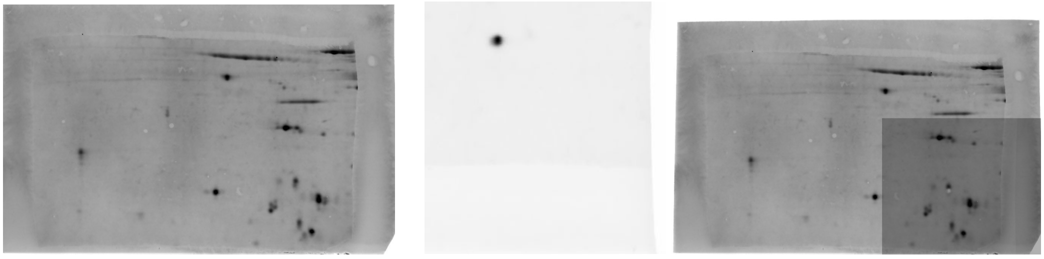

Cy2 signal **Cy5 signal** **Overlay**

Addition to Figure S5: (**a**) 2D-WB of F13A1 83 kDa and (**b**) F13a1 55 kDa.

(**a**)

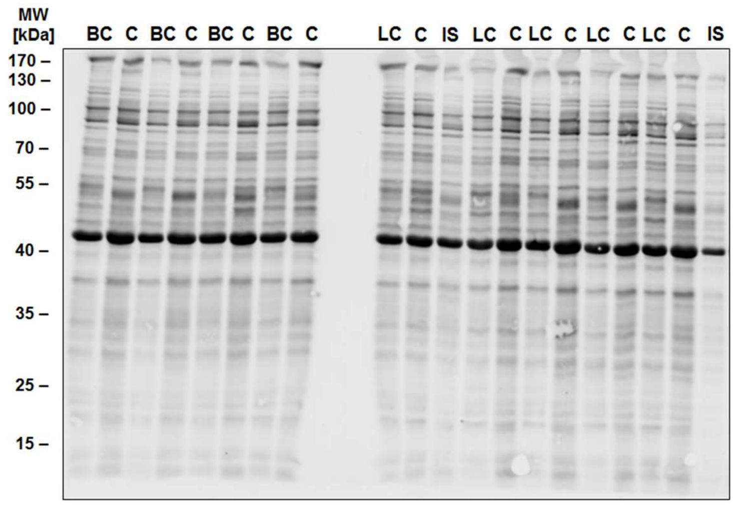

(**b**)

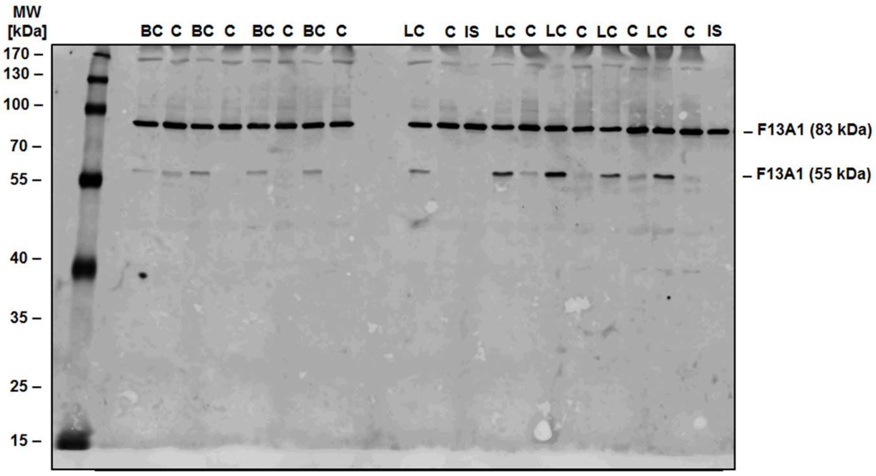

Addition to Figure S6: (**a**) Ruthenium-based whole-protein stain of blotted proteins and (**b**) F13A1 antibody signals detected by fluorescence signal (Cy5).

(**a**)

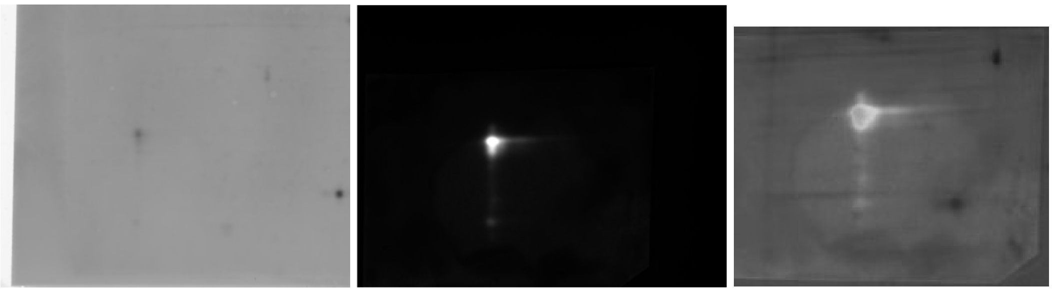

Cy2 signal **Cy5 signal** **Overlay**

(**b**)

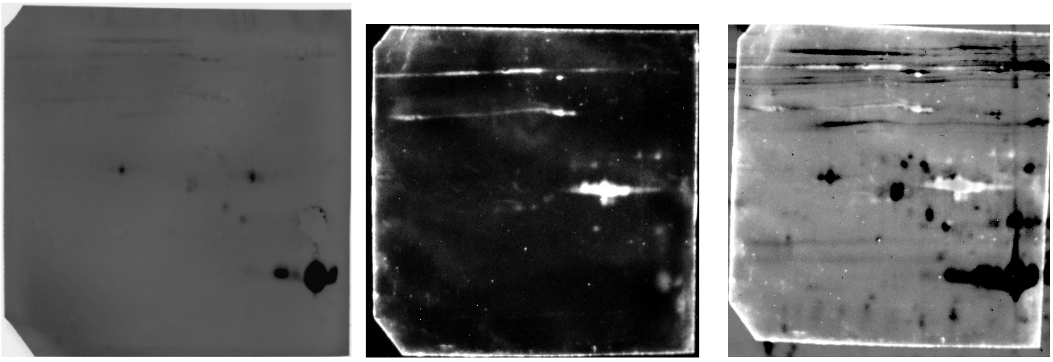

Cy2 signal **HRP signal** **Overlay**

(**c**)

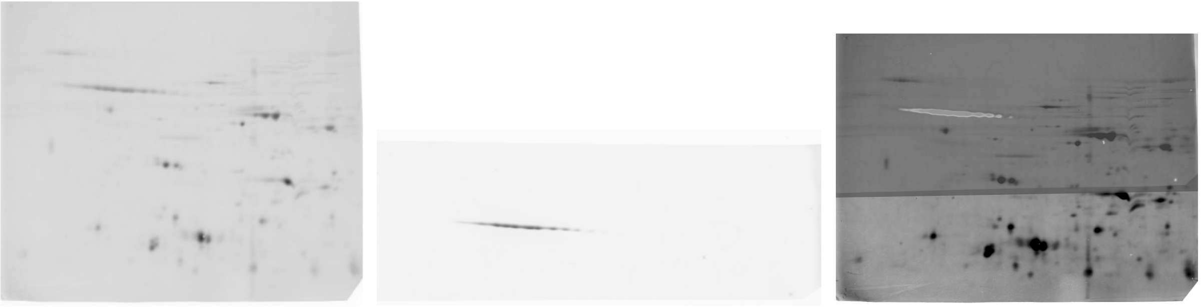

Cy2 signal **Cy5 signal** **Overlay**

(**d**)

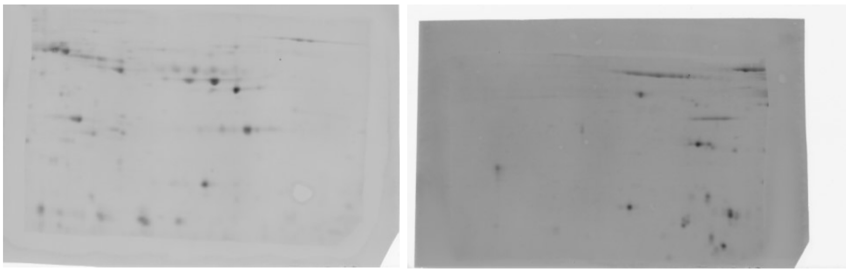

Cy2 signal **Cy2 signal**

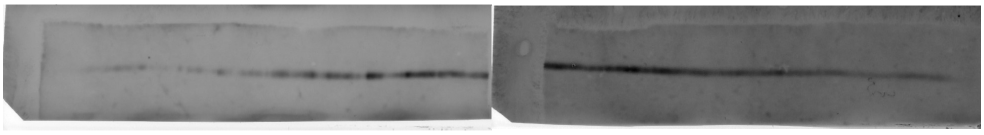

Cy5 signal **Cy5 signal**

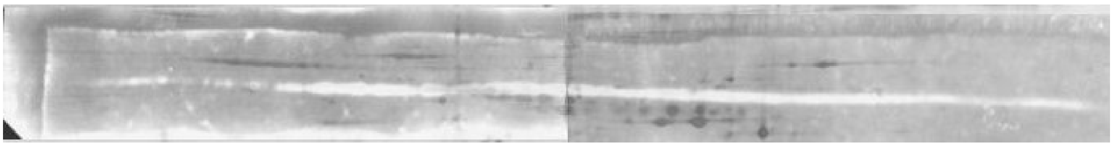

Overlay

(**e**)

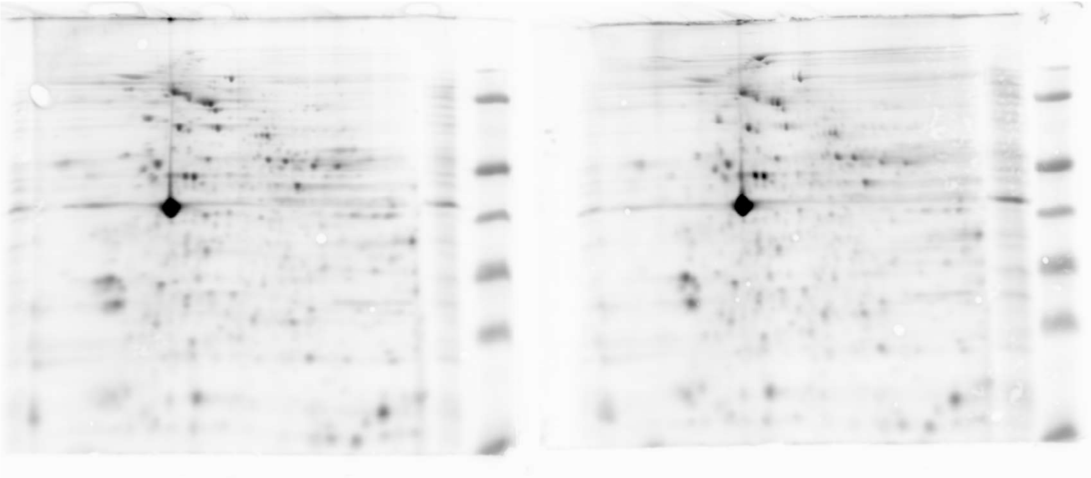

Cy5 signal

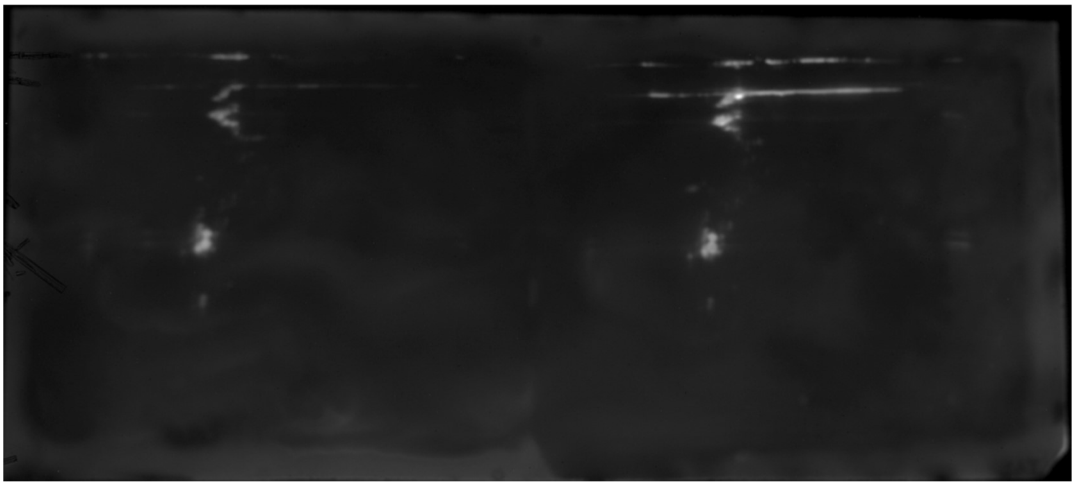

HRP signal

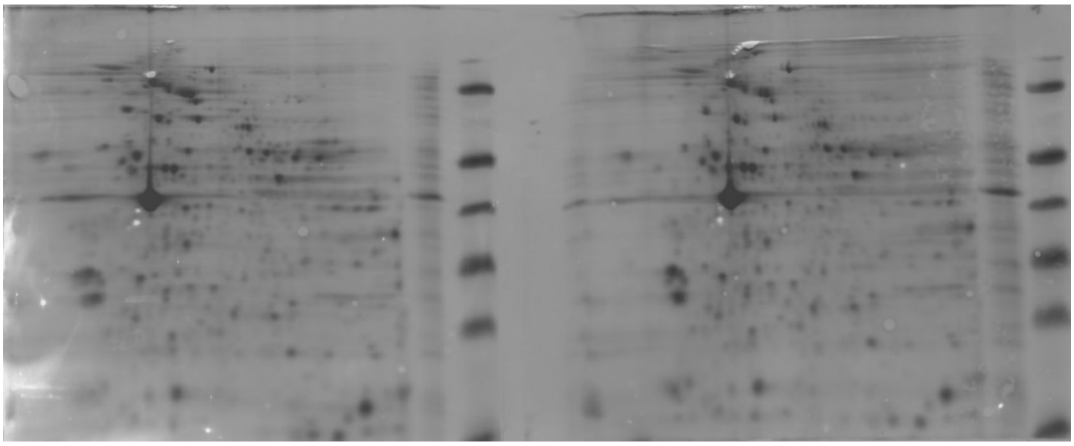

Overlay

Addition to Figure S7: (**a**) 2D-WB of CALR and HSPA5, (**b**) ITGA2B, (**c**) ITGB3, (**d, e**) TLN1.
